# DEK protein level is a biomarker of CD138^positive^ normal and malignant plasma cells

**DOI:** 10.1371/journal.pone.0178025

**Published:** 2017-05-30

**Authors:** Zihni Onur Çalışkaner, Türkan Çakar, Emrah Özçelik, Ahmet Özdilek, Annette S. Kim, Öner Doğan, Amma Bosompem, Gerard Grosveld, Bülent Saka, Ayten Kandilci

**Affiliations:** 1 Department of Molecular Biology and Genetics, Gebze Technical University, Gebze, Kocaeli, Turkey; 2 Department of Pathology, Microbiology and Immunology, Vanderbilt University School of Medicine, Nashville, Tennessee, United States of America; 3 Department of Pathology, Istanbul University, Istanbul Medical Faculty, Istanbul, Turkey; 4 Department of Genetics, St Jude Children’s Research Hospital, Memphis, Tennessee, United States of America; 5 Department of Internal Medicine, Istanbul University, Istanbul Medical Faculty, Istanbul, Turkey; University of South Alabama Mitchell Cancer Institute, UNITED STATES

## Abstract

Overexpression of *DEK* oncogene is associated with increased proliferation of carcinoma cells and it is observed in several solid tumors due to the amplification of the 6p22.3 chromosomal region where *DEK* locates. Although the same chromosomal amplification occurs in multiple myeloma (MM), a plasma cell neoplasm, whether the expression and the copy number of the *DEK* gene are affected in MM remains elusive. We show that despite the increased copy number in CD138^positive^ MM cells (4 out of 41 MM samples), *DEK* mRNA expression was down-regulated compared with that in CD138^negative^ bone marrow (BM) cells of the same patients (P<0.0001). DEK protein was not detectable by immunohistochemistry (IHC) in CD138^positive^ normal plasma cells or in malignant plasma cells of MM patients (n = 56) whereas it was widely expressed in normal and neoplastic B-cells. Stable knockdown or overexpression of DEK in CD138^positive^ MM cell lines did not affect the proliferation and viability of the cells profoundly in the presence or absence of chemotherapeutic agent melphalan whereas knockdown of DEK moderately but significantly increased the expression level of *CD138* (p<0.01). Decreased DEK expression in plasma cells suggests a potential role of this gene in plasma cell development and lack of detectable DEK protein by IHC could be used as a biomarker for normal and malignant plasma cells.

## Introduction

Multiple myeloma (MM) is a malignancy characterized by invasion of the bone marrow (BM) and bones with abnormal plasma cells that are expanded clonally [1, 2]. Cytogenetically, aberrations in MM can be divided into those carrying balanced translocations typically involving the immunoglobulin heavy chain gene and those carrying numerical changes. The latter often involve trisomies but may comprise recurrent deletions or gains of subchromosomal material as well, including gains of 6p22.3-p21.3, found in about 16% of MM patients [3, 4].

The *DEK* oncogene, located on 6p22.3, was initially identified in acute myeloid leukemia as a partner of the *DEK-CAN* fusion gene [5]. It encodes a nuclear protein [6], which is highly expressed in proliferating cells, and it participates in several cellular processes, including chromatin modeling and inhibition of senescence [7, 8]. *DEK* expression is upregulated, most commonly in association with amplification of the genetic locus, in several solid tumors including breast cancer [9, 10], melanoma [11], bladder cancer [12], and retinoblastoma [13]. Consistently, *DEK* overexpression transforms epithelial cells and promotes cancer in mouse models, whereas *DEK* knockdown induces cell death in tumor cells but not in differentiated epithelial cells [14]. Although *DEK* has been shown to contribute to myeloid differentiation of hematopoietic stem/precursor cells and cell lines [11, 15, 16], it remains to be determined whether its expression affects the biology and function of normal and neoplastic plasma cells, especially in the context of 6p amplification.

Here we determined the expression level and copy number of the *DEK* gene in MM cells. To this end, we used formalin fixed paraffin embedded (FFPE) BM samples as well as CD138^positive^ (malignant plasma cells) and CD138^negative^ cells isolated from fresh or frozen BM samples of MM patients and analyzed the copy number and expression level of the *DEK* gene using qPCR and RT-qPCR, respectively. IHC analysis with antibodies against DEK and CD138 was performed on the FFPE samples of MM and monoclonal gammapathies of uncertain significance (MGUS) patients, the latter of whom carry a risk of progression to symptomatic MM of approximately 1% per year. Additional IHC analysis was also performed on the FFPE samples of Burkitt lymphoma (BL), mantle zone lymphoma (MZL) and diffuse large B cell lymphoma (DLBCL) patients. Finally, we stably knocked-down or overexpressed DEK in MM cell lines to determine if change in DEK expression influences the expression level of *CD138* and the growth of MM cells in the presence or absence of the chemotherapy agent melphalan.

## Materials and methods

### Patient samples

FFPE BM tissues of patient samples were obtained from Vanderbilt University (MM (n = 26), MGUS (n = 12), and control BM (n = 9), BL (n = 3), MZL (n = 7) and DLBCL (n = 12)) and Istanbul University, Istanbul Medical Faculty, Department of Pathology (MM (n = 30), control BM (n = 9)). CD138^positive^ and CD138^negative^ cells were isolated from 41 fresh/frozen BM samples of MM patients (Vanderbilt University), 12 of which were obtained concurrently with the FFPE samples listed above. All samples were obtained at diagnosis. The stage of disease was determined by Durie-Slamon criteria [17]. The study was approved by the Institutional Review Boards of Vanderbilt University and Istanbul University and informed consent was obtained from patients in accordance with the Declaration of Helsinki.

### Isolation of CD138^positive^ and CD138^negative^ cells

CD138^positive^ and CD138^negative^ cells from frozen or fresh BM samples were isolated using the EasySep^™^ CD138 positive selection kit (Stem Cell Technologies, Vancouver, BC) according to the manufacturer’s instructions. Purity of the isolated CD138^positive^ population was confirmed by fluorescence activated cell sorting (FACS).

### RNA isolation and RT-qPCR

Total RNA isolation was performed by using the Ambion RecoverAll^™^ Total Nucleic Acid Isolation Kit (Life Technologies, Grand Island, NY) and cDNA synthesis was performed using the High Capacity cDNA Reverse Transcriptase Kit (Life Technologies, Grand Island, NY) following the manufacturer’s instructions. TaqMan, Applied Biosystems primer probes for *ABL1* (Hs01104728_m1) and/or human *ACTB* (Hs01060665_g1) were used to normalize the expression of human *DEK* (Hs00180127_m1) or *CD138* (Hs00896423_m1). Relative *DEK* expression was calculated using three pooled healthy BM samples as a calibrator based on the 2^[-ΔΔCT]^ method or by using a standard curve method. Samples with a cycle time (Ct) value ≤ 40 were deemed positive for the expression of the analyzed gene. All reactions were performed in triplicate.

### Determination of the *DEK* gene copy number

DNA was isolated using the QIAamp DNA Blood Mini Kit (Qiagen, Valencia, CA) and qPCR was performed in quadruplicate using 20 ng DNA and primer probe sets for *RNase P* and *DEK* (Applied Biosystems, 4403326 and Hs04904663_cn, respectively) following the manufacturer’s instructions. qPCR values of the *DEK* gene were normalized against those of *RNase P* (2 copies per genome) and a relative copy number of *DEK* was calculated using control BM DNA (three pooled samples) as a calibrator based on the 2^[-ΔΔCT]^ method. Normalized qPCR values of control, carrying two gene copies, were scored as “1”, while values of patient samples ≥1.5 fold or ≤0.5 fold were considered to represent an increased or decreased gene copy number, respectively.

### Immunohistochemical (IHC) and immunofluorecent (IF) staining

After the antigen retrieval (45 minutes in Borg Decloaker RTU (Biocare Medical, Concord, CA) in a steam cooker) slides were cooled to room temperature (20 min) in the same buffer, washed with TBS (ScyTek, Logan) and incubated for 15 min with 3% H_2_O_2_ (in distilled water) (Sigma-Aldrich, St. Louis, MO). Slides were stained with anti-Human DEK antibody (1:60 diluted; 60 min) (BD Biosciences, San Jose, CA) and an HRP-conjugated secondary antibody (30 min) (Biocare Medical), followed by 3,3'Diaminobenzidine (DAB) chromogen (Biocare Medical) incubation for 10 min. For double IHC staining, the same slides were denatured for 5 min with a denaturing solution (Biocare Medical), followed by incubation with anti-human CD138 antibody (1:400 dilution; 60 min) (Biocare Medical), alkaline phosphatase (AP)-conjugated secondary antibody (30 min) (Biocare Medical), and Warp-red chromogen (Biocare Medical) (10 min), respectively. All the incubations were done at room temperature and slides were washed (3x5min) with Tris-buffered saline between each step. Slides were counterstained with Gill’s Hematoxylin (3 min) (American MasterTech, Lodi, CA).

IF staining of RPMI-8226 cells was performed as described [18]using CD138 antibody (1:400 dilution; 60 min) and Alexa-555 labeled mouse anti human secondary antibody (1:400 dilution; 60 min) (Cell Signaling Technologies (CST), Danvers, MA). CD138 expression was also analyzed by FACS using PE-labeled mouse anti human CD138 antibody (BD Biosciences) as described [19].

### Western blot analysis

Total cell lysates were prepared in RIPA buffer (150 mM NaCl, 1% NP40, 0.5% Sodium Deoxycholate, 0.1% SDS, 50 mM Tris-HCl, pH:8) containing protease and phosphatase inhibitors (ThermoFisher Scientific, Asheville, NC) followed by sonication (2 x 15 seconds). The lysates were separated on a 10% TRIS-HCL polyacrylamide gel (Biorad, Hercules, CA) and transferred to nitrocellulose membranes (Biorad). The membranes were incubated overnight at 4°C with mouse anti-human DEK (1:1000) (BD Biosciences) or with rabbit anti human GAPDH (1:5000) (CST) for 1 hour at room temperature, followed by incubation with horseradish peroxidase (HRP) conjugated secondary antibodies (CST). Protein bands were visualized with chemiluminescence (ThermoFisher Scientific,) using the ChemiDoc imaging system (Biorad).

### Virus preparation and transduction

Full-length human *DEK* cDNA was generated from human CD34^positive^ cells and cloned into the EcoRI site of MSCV-IRES-GFP (MIG) retroviral vector. *DEK* primers used for cDNA preparation were as follows: forward 5’-ATGTCCGCCTCGGCC-3’, reverse 5’-TCAAGAAATTAGCTCTTTTACAG-3’. The integrity of the cDNA was confirmed by sequencing. VSVg peudotyped retrovirus was prepared using MIG and MIG-DEK constructs as described [19]. A scrambled non-targeting (Origene, Rockville, MD, TR30021) and DEK-specific short hairpin (sh)-lenti viral constructs (Origene, TL313507) were used to prepare VSVg pseudotyped third generation lentivirus [19]. Transduced cells were propagated and GFP^positive^ cells were sorted using FACS. Constructs that gave the greatest knockdown of DEK (Origene, TL313507B (shDEK-B), TL313507C (shDEK-C)) were used in further experiments.

### Cell lines, growth curve and cell cycle analysis

U266 (TIB-196) or RPMI-8226 (CCL-155) cells were obtained from ATCC (Manassas, VA) and maintained in RPMI-1640 containing glutamine (Gibco, ThermoFisher Scientific, 52400025, MA, USA), 15% fetal bovine serum (Gibco, 10270106), and 1% penicillin/streptomycin (Gibco, 15140122). For cell cycle and growth curve analyses, FACS-sorted GFP^positive^ cells were seeded (2x10^5^ cells/ml) into 6-well plates in 3ml medium in the presence of 10 or 20 μM melphalan or vehicle (acid alcohol), in duplicate daily, and the total cell number was counted using a Countess cell counter (Invitrogen, ThermoFisher Scientific, C10227). Viability was calculated for each analyzed time point (viable cell number/total cell number x 100) and the percentage of viable cells was represented relative to the viability at “time zero”. Cell cycle analysis was performed using propidium iodide staining (PI) and FACS.

### Statistical analysis

Statistical analyses were performed using GraphPad Prism, version 5.0 for Windows (GraphPad Software; www.graphpad.com). P values lower than 0.05 were considered significant.

## Results

### Total BM samples of MM patients show decreased *DEK* mRNA expression

**Fig 1:**
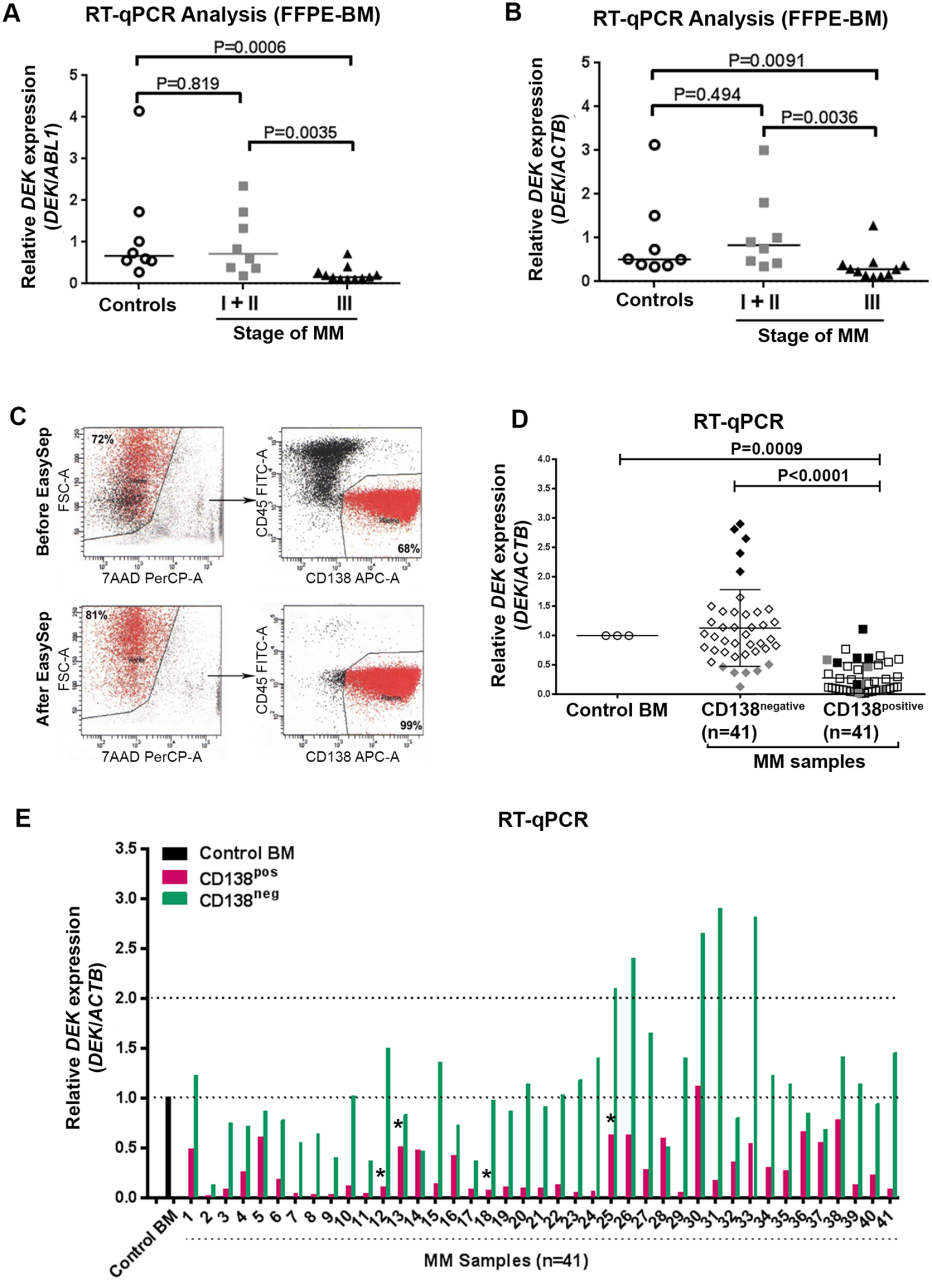
*DEK* expression in MM cells. (A, B) RT-qPCR and Mann-Whitney statistical analysis of *DEK* expression (average of triplicates) in FFPE total BM samples of healthy controls (n = 8) and MM patients. *DEK* expression was normalized against *ABL1* (A) and *ACTB* (B) expression. (C) FACS analysis of purified CD138^positive^ cells in one representative patient sample (Table 2, patient number 25) showing the purity of the isolated cells. (D, E) RT-qPCR and Mann-Whitney statistical analysis of *DEK* expression in control total BM (3 pooled healthy samples) and CD138^positive^ and CD138^negative^ cells of MM patients isolated from fresh or frozen BM samples. Black or grey diamonds (D, middle column) indicate samples with ≥ 2-fold increased or decreased *DEK* expression in CD138^negative^ cells, respectively. Black or grey squares (D, right column) represent the *DEK* expression in CD138^positive^ cells of the samples corresponding to black or grey diamonds described above, respectively. Asterices (E, samples 12, 13, 18, 25) indicate the samples with copy number gains of the *DEK*.

To determine the expression level of *DEK* mRNA in MM patients, we first analyzed the archival FFPE BM samples from MM patients and healthy controls using RT-qPCR. Using two different house-keeping genes (*ACTB* and *ABL1*) as normalizer [20, 21] we found that although the expression level of *DEK* was similar between the control BM (n = 8) and patient samples with stage-I (n = 1) and stage-II (n = 7) MM (Fig 1A and 1B), there was no detectable *DEK* mRNA expression in 10 out of 21 patients with stage-III MM (Table 1). Moreover, when the patients with stage-III MM lacking *DEK* expression were excluded from the data, expression levels of *DEK* in the rest of the stage-III patient samples was significantly lower than those of healthy controls (P = 0.0006 (for *DEK/ABL1*) and P = 0.0096 (for *DEK/ACTB*)) and patients with stage-I/stage-II MM (P = 0.0035 for *DEK/ABL1* and P = 0.0036 for *DEK/ACTB*)) (Fig 1A and 1B). These data suggested that *DEK* expression is down regulated in the BM of stage-III MM patients.

**Table 1:** Pathological feauters and normalized *DEK* expression levels of FFPE samples of controls (1–8) and MM patients (9–38).

No	Gender	Age	Stage	Pathology	Relative *DEK* Expression	
					*DEK/ACTB*	*DEK/ABL1*
1	M	73	N/A	Control BM	3.13	4.15
2	M	73	N/A	Control BM	1.51	1.73
3	M	65	N/A	Control BM	0.51	0.55
4	M	45	N/A	Control BM	0.39	0.60
5	F	57	N/A	Control BM	0.35	0.56
6	M	78	N/A	Control BM	0.52	1.02
7	M	62	N/A	Control BM	0.37	0.28
8	F	36	N/A	Control BM	0.74	0.74
9	M	69	2A	Int. Nod.	0.35	0.39
10	F	80	1A	Int.	1.00	0.84
11	F	73	2A	Int.	0.76	0.60
12	F	72	2A	Int.	0.91	2.35
13	F	64	2A	Nod.	1.81	1.72
14	F	76	2A	Int.	0.42	0.19
15	M	64	2A	Diffuse	3.01	1.33
16	M	58	2A	Diffuse	0.47	0.37
17	M	54	3A	Nod. Dif.	0.28	0.11
18	F	51	3A	Int.	0.37	0.10
19	F	57	3A	Int.	0.23	0.28
20	M	57	3A	Int.	0.00^a^	0.00^a^
21	F	60	3A	Int. Dif.	0.00^a^	0.00^a^
22	F	58	3A	Int.	0.12	0.15
23	M	61	3A	Int.	0.16	0.20
24	M	72	3A	Diffuse	0.29	0.11
25	F	66	3A	Diffuse	0.00^a^	0.00^a^
26	F	51	3B	Diffuse	0.00^a^	0.00^a^
27	M	50	3B	Nod.	0.00^a^	0.00^a^
28	M	49	3B	Diffuse	1.28	0.72
29	M	60	3A	Int.	0.00^a^	0.00^a^
30	M	76	3B	Int.	0.00^a^	0.00^a^
31	F	64	3B	Int.	0.10	0.13
32	F	63	3B	Int.	0.14	0.16
33	M	54	3B	Nod. Int.	0.00^a^	0.00^a^
34	M	55	ND	Diffuse	0.44	0.21
35	F	62	3A	Int.	0.39	0.41
36	M	62	3B	Int.	0.00^a^	0.00^a^
37	F	78	3A	Int.	0.00^a^	0.00^a^
38	M	75	3A	Diffuse	0.00^a^	0.00^a^

Int.: Interstitial; Dif.: Diffuse; Nod.: Nodular; N/A: Not applicable.

^a^ Undetectable *DEK* expression (Ct ≥40).

### *DEK* mRNA expression is reduced in CD138^positive^ plasma cells in MM

We hypothesized that the observed decrease in *DEK* expression in BM samples of MM patients reflects the level of *DEK* in malignant plasma cells, since the CD138^positive^ neoplastic plasma cell (aka MM cells) burden in these marrows was increased (greater than 10% of the total cellularity). To test this hypothesis, CD138^positive^ and CD138^negative^ cells were isolated (Fig 1C) from the fresh or frozen BM samples of MM patients (n = 41) and examined using RT-qPCR. Consistent with the FFPE RT-qPCR results, we found an overall statistically significant decreased *DEK* expression in the CD138^positive^ cells compared to both CD138^negative^ cells of the same patients (P<0.0001) and to the control total BM cells (P = 0.0009) (Fig 1D). *DEK* expression was lower in the CD138^positive^ MM cells in 39 out of 41 samples compared with CD138^negative^ cells of the same patients. In 34 of 41 samples (82%) there was between a 2 to 30-fold decrease in expression of *DEK* in CD138^positive^ cells compared to the corresponding CD138^negative^ cells, whereas 12% of the samples (5/41) showed between 1.3 to 1.7-fold decrease in *DEK* expression (Table 2) (Fig 1E). As expected, expression levels of *DEK* in the CD138^negative^ cells of 73% of MM patients (30 out of 41) was similar to that in the control total BM sample, whereas 6 out of 41 MM samples showed ≤2 fold decrease and 5 out of 41 samples showed ≥2 fold increase in *DEK* expression in CD138^negative^ cells (Table 2) (Fig 1E). Together, these data indicated that *DEK* mRNA expression was significantly reduced in CD138^positive^ MM cells.

**Table 2:** Molecular and clinical features of the fresh or frozen patient (MM) samples (n = 41).

ID	Gender	Age	*DEK* RTq-PCR		*DEK* Copy number		Plasma cell (%) at diagnosis	IHC
			CD138^p^	CD138^n^	CD138^p^	CD138^n^		
1	F	74	0.48	1.23	1		38.5	
2	M	48	0.01	0.13	1.09		90	
3	M	60	0.08	0.75	1.05		27.5	
4	F	72	0.25	0.72	1.04		61	
5	F	75	0.60	0.87	1.03		45	
6	F	63	0.18	0.78	1.16		21	
7	F	N/A	0.04	0.55	1.16		78	
8	M	70	0.02	0.64	1.03		64.5	
9	M	69	0.03	0.40	0.86		40	
10	M	49	0.11	1.02	1.02		21.5	
11	M	57	0.04	0.37	1.21		17	(+)
12	M	66	0.10	1.50	**1.78**	**1.02**	22.5	(+)
13	M	65	0.50	0.83	**1.55**	**1.02**	33	(+)
14	M	56	0.47	0.47	1.09		55.5	
15	F	59	0.13	1.36	1.03		41	
16	M	61	0.42	0.73	0.92		30	
17	M	59	0.08	0.37	1.17		21.5	
18	F	69	0.07	0.98	**1.72**	**1.06**	32.5	(+)
19	M	63	0.10	0.87	1.44		41.5	
20	N/A	N/A	0.09	1.14	0.88		22	
21	M	67	0.09	0.91	1.34		13	
22	N/A	59	0.12	1.03	0.96		52	
23	F	51	0.05	1.18	1.01		32.5	
24	F	51	0.06	1.40	1		52.5	
25	F	68	0.62	2.09	**1.96**	**1.12**	60	(+)
26	F	71	0.62	2.40	1.1		20	(+)
27	F	74	0.27	1.65	0.82		23	
28	M	87	0.59	0.51	0.67		7	
29	M	75	0.05	1.40	1.02		35	(+)
30	M	79	1.11	2.65	1.07		54	(+)
31	M	53	0.17	2.90	0.91		10	(+)
32	M	N/A	0.35	0.80	0.92		5	
33	M	67	0.54	2.81	0.96		10	(+)
34	M	48	0.30	1.23	1.18		10	(+)
35	F	52	0.26	1.14	0.89		25	
36	M	68	0.66	0.85	0.81		23	(+)
37	M	62	0.55	0.68	1.03		20	
38	M	57	0.77	1.41	0.88		6	
39	F	63	0.12	1.14	1.05		46.5	
40	M	51	0.22	0.94	1.02		15	
41	M	66	0.08	1.45	1.24		21	

^p^ Positive;

^n^ Negative;

N/A: Not applicable.

Note: ID numbers 23 and 24 represents two different samples obtained from the same patient at different time points.

### Copy number changes of the DEK gene in MM cells

**Fig 2:**
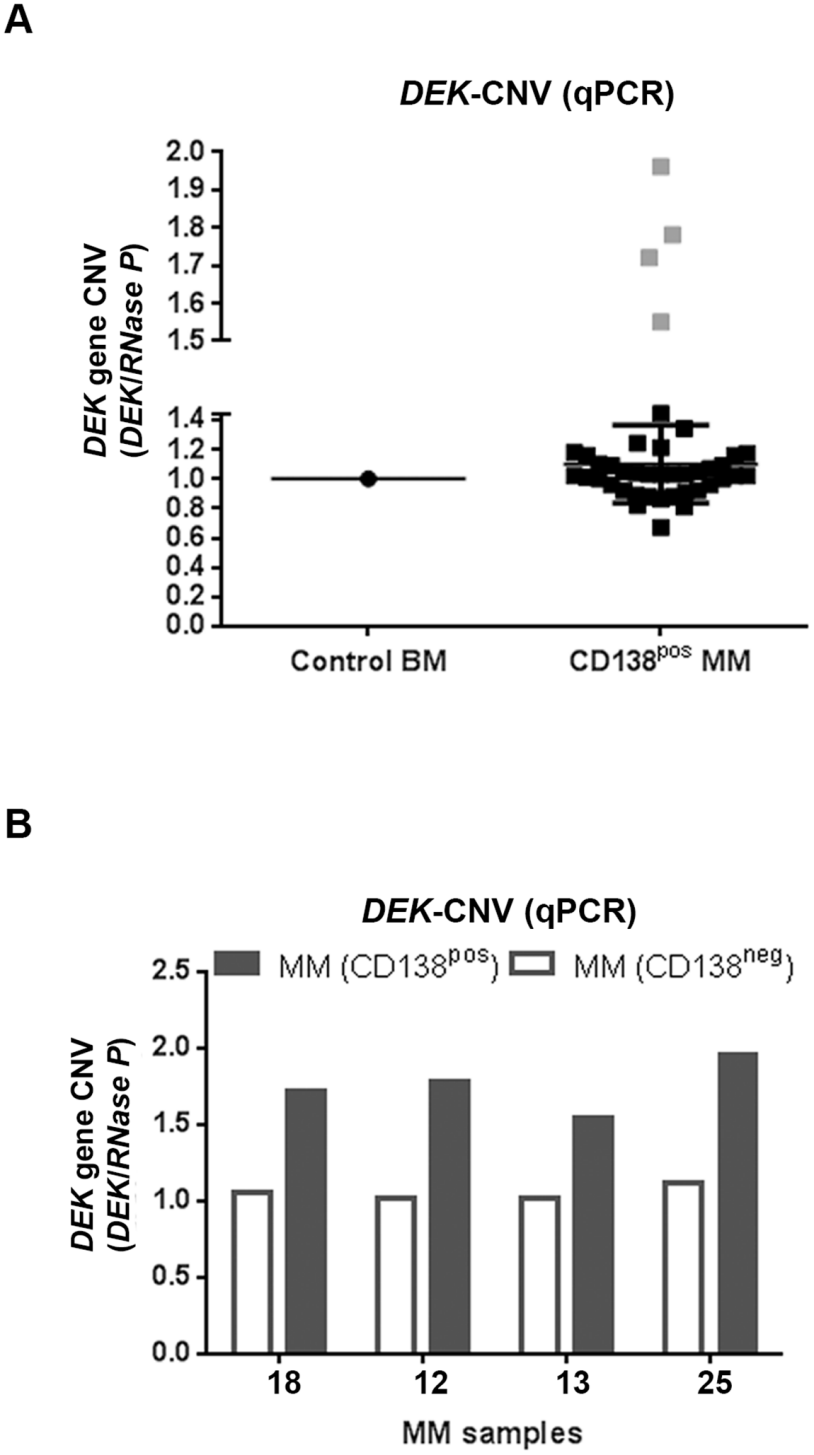
Copy number variation (CNV) of the *DEK* gene in CD138^positive^ cells. (A) qPCR CNV analysis of the *DEK* gene, showing the average of four replicates per sample, in control BM (3 pooled samples) and CD138^positive^ MM cells (n = 41). The MM samples with *DEK* amplification are shown in grey squares. (B) qPCR CNV analysis of the CD138^negative^ cells of the patients showing *DEK* amplification in the CD138^positive^ MM cells (shown in grey squares in the Fig 2A).

Given that gains of the chromosome region 6p22.3 is observed in 16% of MM patients [4], we hypothesized initially that the *DEK* gene would be amplified and overexpressed in MM patients who carry 6p22.3 amplification, as observed in the other solid tumors [12, 13]. To our surprise, none of the patient samples in our cohort showed *DEK* overexpression in CD138^positive^ MM cells (Fig 1E) suggesting that either *DEK* was not amplifid or its amplification did not cause overexpression in the CD138^positive^ MM cells. To distinguish between these possibilities, we analyzed the copy number variation (CNV) of the *DEK* gene (DEK-CNV) in the CD138^positive^ cells of these 41 samples using a qPCR assay. We found that the *DEK* allele was amplified in the CD138^positive^ MM cells in 4 of 41 samples (≥1.5 fold, 3 copies or more) compared with the control DNA obtained from total BM samples of healthy donors (3 pooled DNAs) (Fig 2A and Table 2). Moreover, *DEK* amplification was specific to the CD138^positive^ cells in these samples while the CD138^negative^ cells of the same patients contained two copies of the *DEK* gene (Fig 2B and Table 2). Interestingly, amplification of the *DEK* gene in the CD138^positive^ MM cells did not increase the expression level of *DEK* mRNA. Compared to paired CD138^negative^ cells from the same patients, *DEK* was down-regulated in these CD138^positive^ MM cells by 15, 1.6, 14, and 3.3-fold, respectively (Fig 1E and Table 2, patient numbers 12, 13, 18 and 25 marked by asterices). These results suggest that *DEK* mRNA expression is decreased in terminally differentiated plasma cells, regardless of the copy number of this gene.

### Lack of detectable DEK expression by IHC in normal and malignant CD138^positive^ plasma cells

**Fig 3:**
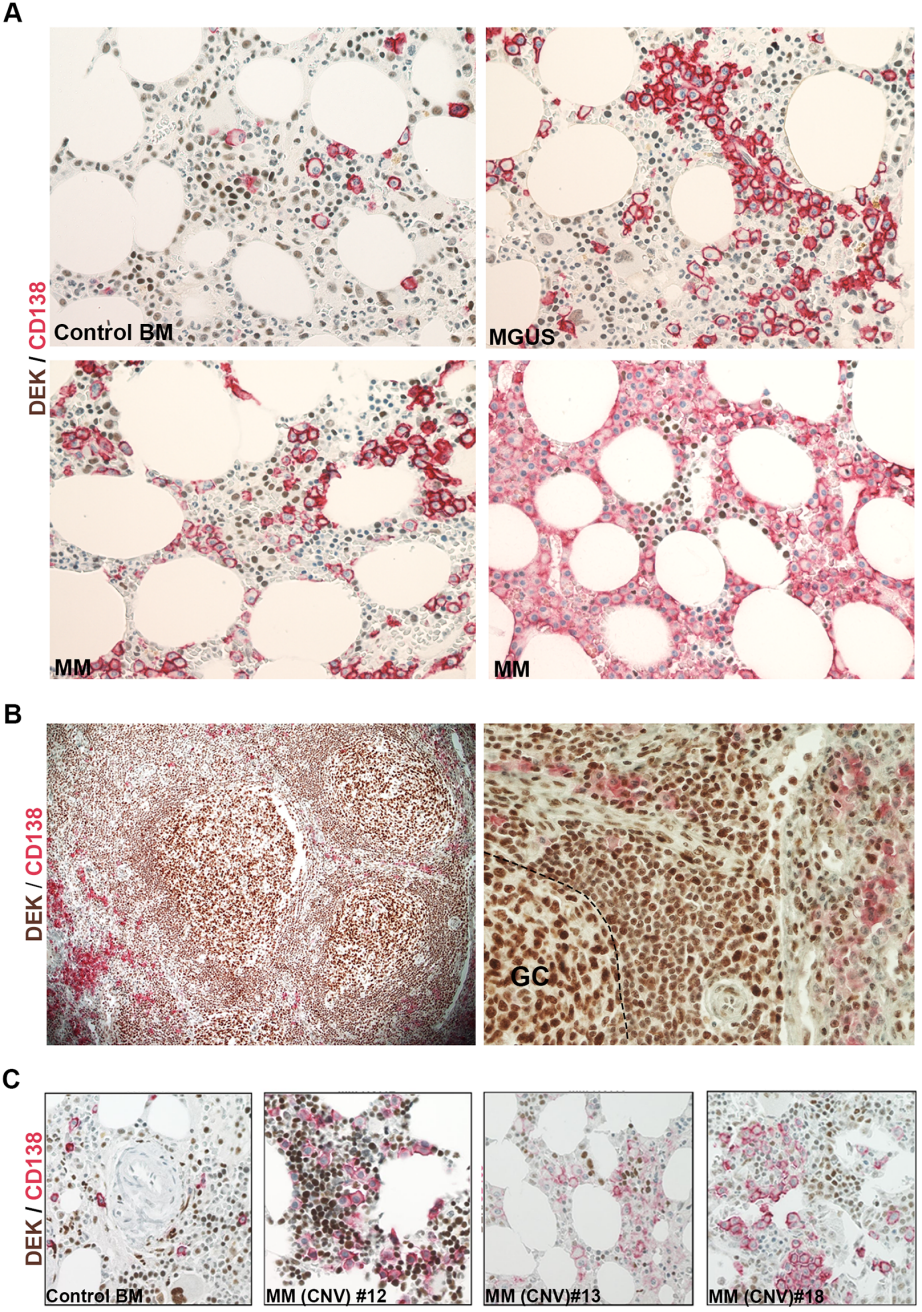
IHC analysis of DEK and CD138 in MM samples. Micrograph of the representative FFPE BM samples of 1 control (Control BM), 1 MGUS and 2 different MM patients (MM; lower left and right panels) (400X) (A); a proliferative lymph node used as a control (Left panel 200X, right panel 400X) (B); and a control BM and 3 MM samples with DEK amplification (400X) (C), revealing the CD138 staining (Red) of the membranes of the plasma cells and DEK staining (Brown) in the other hematopoietic cells by IHC. GC indicates the germinal center of the lymph node. Blue shows nuclear counter staining with hematoxylin.

**Fig 4:**
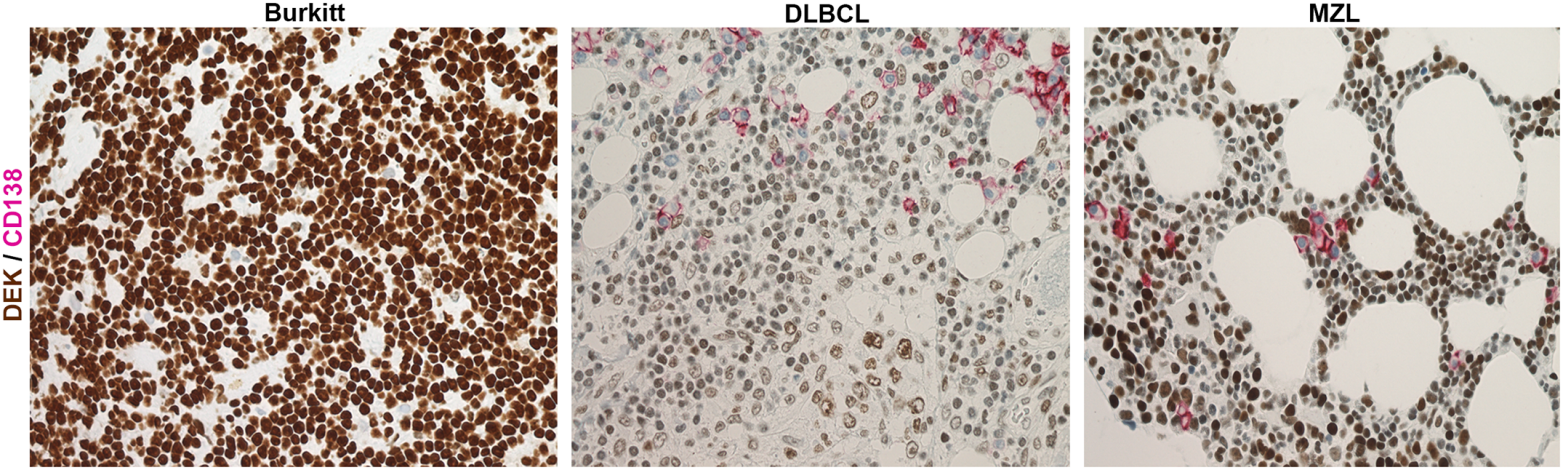
IHC analysis of DEK and CD138 in B-cell malignancies. Micrograph of the representative FFPE samples of BL (left panel, Burkitt), DLBCL (middle panel) and MZL (right panel)(400X) showing DEK (brown) and CD138 (Red) staining. Blue shows nuclear counter staining with hematoxylin.

To determine the expression level of DEK protein in MM cells, we analyzed the FFPE BM samples of MM patients (n = 56) (including the FFPE samples of the 12 out of 41 fresh or frozen samples used in RT-qPCR analysis and 30 BM samples used in FFPE-RT-qPCR analysis) by using either a single or a double IHC staining with antibodies against DEK and CD138. We were not able to perform Western Blot analysis due to limitations in the number of cells that were separated based on CD138 expression. Therefore, we preferred IHC analysis since it is one of the most convenient routine test performed by pathologists for the archived FFPE samples, which provide information about cellular localization and expression level of proteins as well as morphology of the cells. We found a moderate to high level of DEK expression in myeloid and erythroid cells, whereas there was no detectable DEK protein in CD138^positive^ plasma cells in BM samples of controls (n = 8), MGUS or MM patients (Fig 3A). Similarly, IHC analysis of a proliferative lymph node, used as a positive control, showed DEK expression in lymphocytes, particularly in the germinal center cells, but not in the CD138^positive^ plasma cells (Fig 3B, left and right panels). DEK expression was also undetectable in CD138 ^positive^ MM cells of the FFPE samples, which were available from 3 MM patients which showed *DEK* amplification (Fig 3C). Finally, analysis of patients with B cell malignancies, which included BL, MZL and DLBCL, showed similar staining pattern with DEK and CD138 antibodies (Fig 4). These results suggest that the level of DEK expression in mature plasma cells was below the detection limit of the IHC assay and the lack of detectable DEK protein might be an additional useful negative marker for the detection of CD138^positive^ normal and malignant plasma cells.

### Stable knockdown of DEK in MM cell lines moderately increases CD138 expression without a profound effect on the proliferation and viability

**Fig 5:**
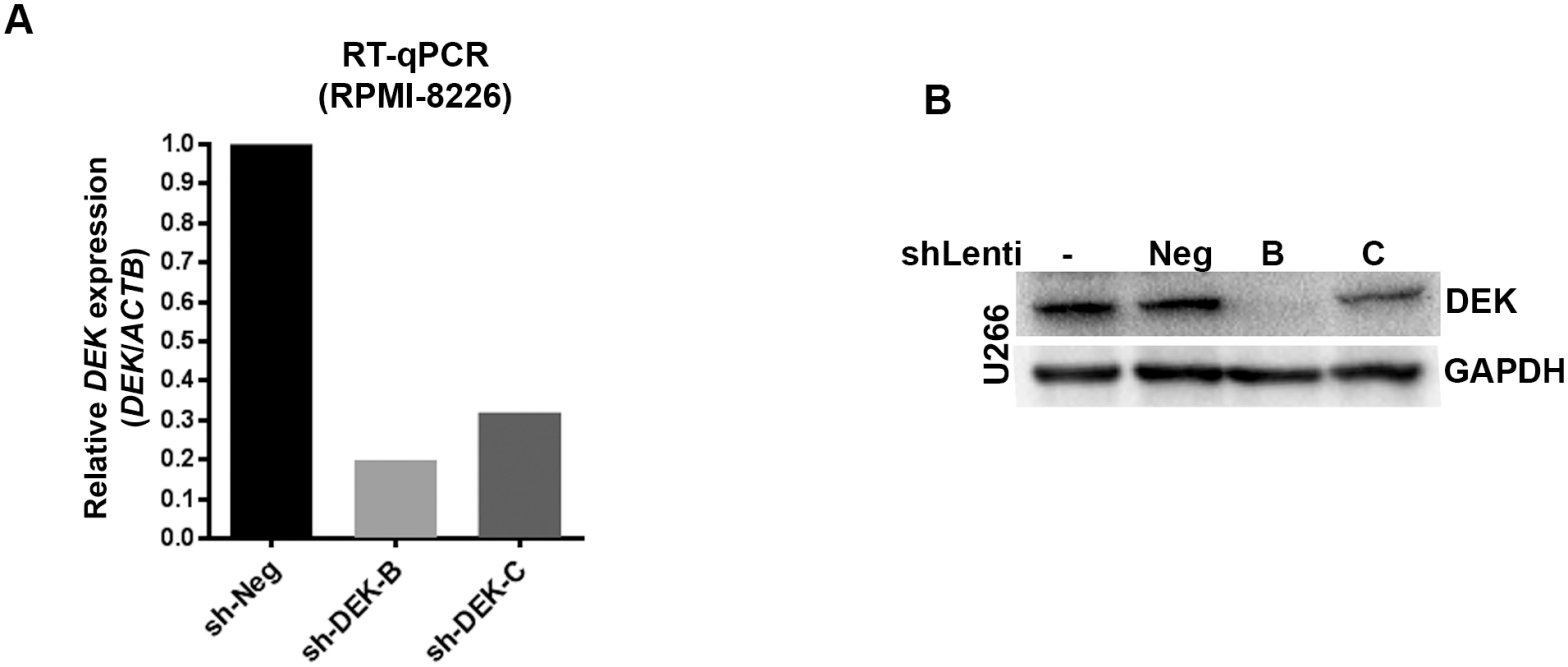
Confirmation of DEK knockdown in MM cell lines. (A, B) RT-qPCR (A) and Western blot analysis (B) of FACS-sorted GFPpositive MM cell lines (RPMI-8226 and U266) showing DEK mRNA and protein expression after the DEK knockdown. Sh-Negative (or Neg) indicates the scrambled non-targeting sh-RNA control.

**Fig 6:**
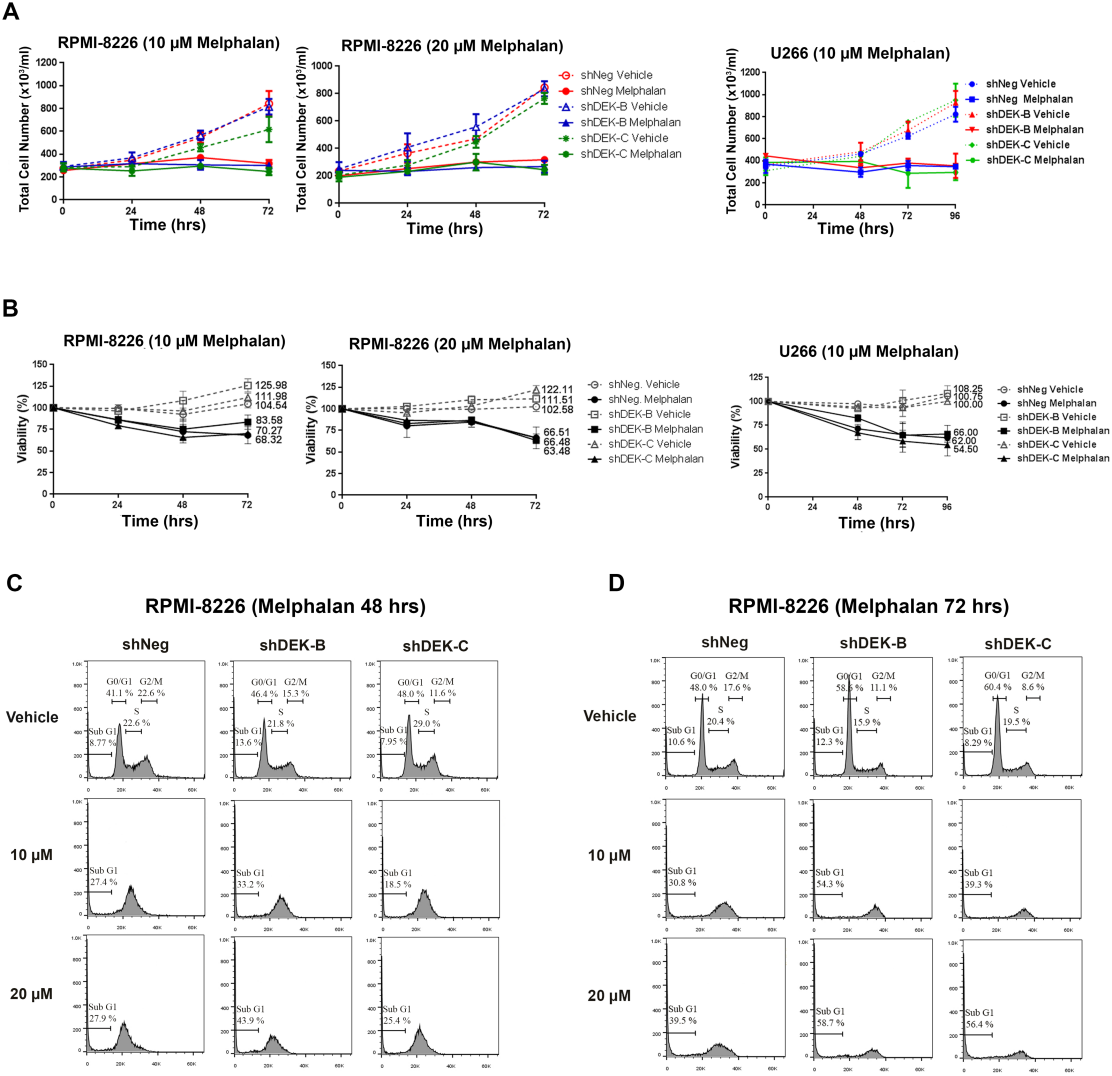
Effect of DEK knockdown on the growth of MM cell lines. (A, B) Growth curve analysis (A) and viability (B) of the RPMI-shDEK and U266-shDEK cells are shown after treatment with the indicated doses of melphalan (solid lines) or vehicle (dotted lines). (C, D) Cell cycle analysis of the same RPMI-shDEK or control RPMI-shNegative cells after 48 (C) and 72 hours (D) of melphalan treatment by using FACS and PI staining.

**Fig 7:**
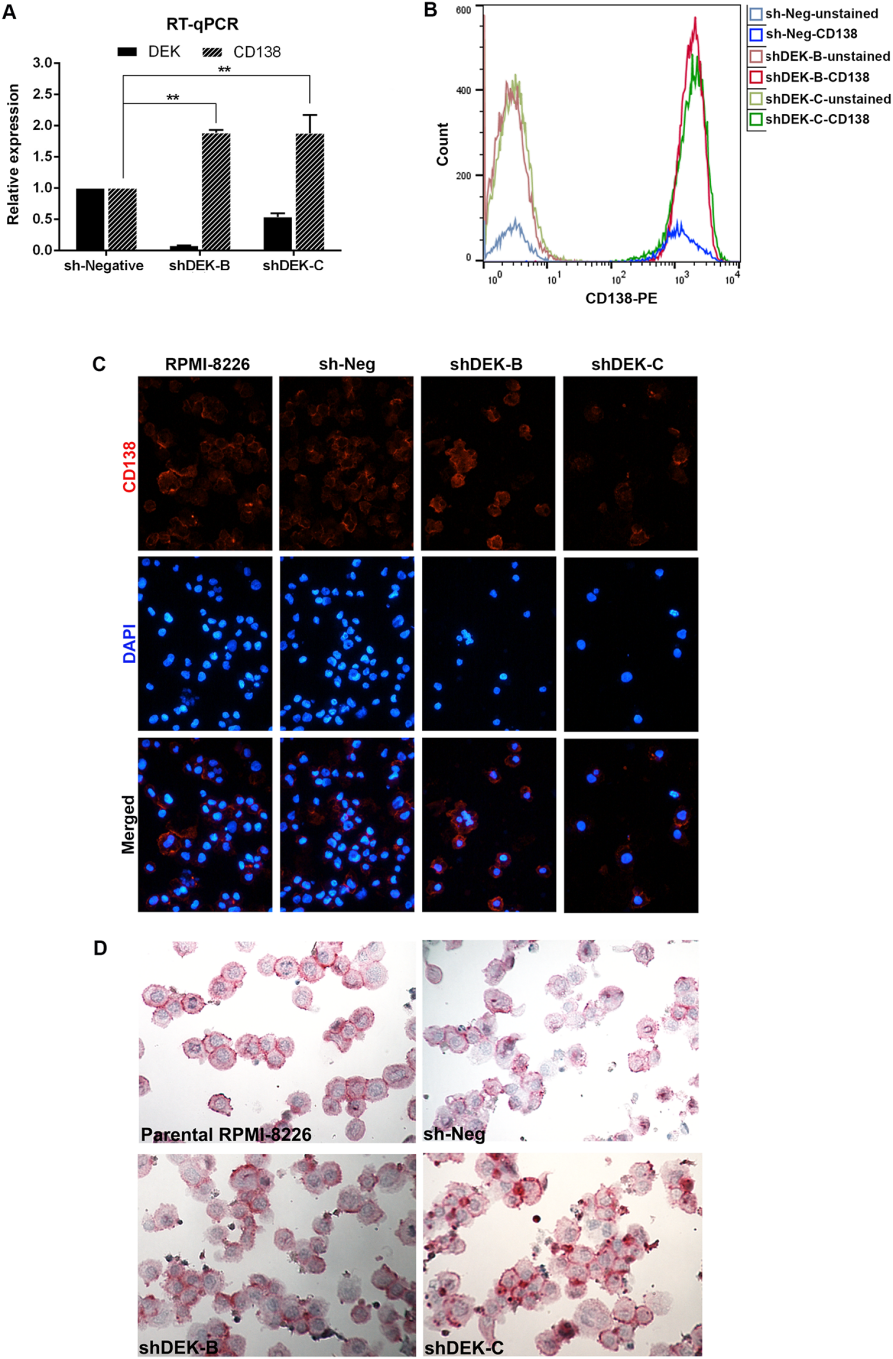
Effect of DEK knockdown on the expression level of CD138. (A) RT-qPCR analysis showing *DEK* and *CD138* expression (average of triplicates) in RPMI-shDEK and control sh-Negative cells. *DEK* expression was normalized against *ACTB* expression (** P<0.01, two-way ANOVA). (B) FACS analysis showing CD138 (CD138-PE) expression in the RPMI-sh-Negative and RPMI-shDEK cells. The lines at the left hand side of the graphic with dim colors indicate the mean fluorescent intensity (MFI) of unstained cells whereas the lines at the right hand side of the graphic with bright colors show the CD138 expression in the same cells stained with CD138-PE. Over 98% of all of the stained cells were positive for CD138. MFI of CD138-stained sh-Negative (bright blue), shDEK-B (bright red) and shDEK-C (bright green) cells was 1352, 1840 and 1893, respectively. (C,D) Micrograph of the parental RPMI-8226 and RPMI-8226-shDEK cells that were subjected to immunofluorescent staining (C) (200X) or immunocytochemical staining (D) (400X) with mouse anti human CD138 antibody.

Next we interrogated the biological effects of altered DEK expression, given that *DEK* was down regulated in primary CD138^positive^ MM cells (Fig 1D and 1E). We stably suppressed *DEK* expression in the MM cell lines RPMI-8226 and U266 using sh-RNA lentiviral constructs targeting *DEK* mRNA. RT- qPCR (Fig 5A) and Western blot analysis (Fig 5B) of transduced and FACS-sorted GFP^positive^ cells confirmed the knockdown with two different shDEK lentiviruses in both cell lines. Growth curve analysis of RPMI-shDEK and U266-shDEK cells in the presence or absence of melphalan, one of the chemotherapeutic agents used in the treatment of MM patients, did not show a significant difference compared with control sh-Negative cells (Fig 6A). Melphalan treatment of the RPMI-shDEK and U266-shDEK cells resulted in a similar level of cell death (Fig 6B) and arrest in the S-G2M phase of the cell cycle in all cases (Fig 6C and 6D). Similarly, overexpression of DEK in both cell lines (FACS-sorted RPMI-DEK-GFP or U266-DEK-GFP) did not change the growth profile or melphalan response of the cells, compared with that of FACS-sorted RPMI or U266 cells transduced with control GFP-only virus (data not shown). Given that *DEK* expression was lower in the primary CD138^positive^ cells, next we tested the expression level of *CD138* in MM cell lines using RT-qPCR and found a moderate (1.8 fold) but significant (P<0.01) increase in the expression level of *CD138* in RPMI-shDEK cells compared to control RPMI-sh-Negative cells (Fig 7A). Flow cytometry (Fig 7B) and immunocytochemical analyses (Fig 7C and 7D) of the same cells revealed a mild increase in CD138 protein on the surface of the RPMI-shDEK cells without a profound effect on the adhesion to fibronectin (data not shown), a process partly mediated by CD138. We did not observe a change in the expression level of *CD138* when we overexpressed DEK in the parental RPMI-8226 cells (data not shown), suggesting a possible indirect association between decreased DEK and increased CD138 expression in RPMI-8226 cells.

## Discussion

Here, we report that *DEK* mRNA and protein expression is decreased in normal plasma cells and MM cells that express CD138 regardless of the level of amplification of the *DEK* gene. This finding is distinct from the concerted findings of *DEK* amplification and overexpression in epithelial cancers.

Due to limitations in our resources, we performed qPCR analysis, a commonly used and accepted technique for large scale analysis of CNV in various diseases [13, 22], to detect the correlation between the CNV and mRNA expression of *DEK*. Despite the relatively small number of MM cases that we have analyzed, we were able to detect a copy number gain in *DEK* gene in 10% of the samples (4/41), which was comparable to the study of Walker et al. showing a gain in the chromosome 6p22.3-p21.31 region, where *DEK* locates, in 16% of MM samples (19/114) using a high-resolution single nucleotide polymorphism mapping array [4]. High level of *DEK* expression is associated with increased cellular proliferation in the epithelial cells [11, 13, 23, 24] and B-lymphoid cells [25]. Consistently, our IHC analysis of a proliferative lymph node indicated high level of DEK expression in the mantle zone and germinal center B cells (Fig 3B), suggesting that DEK expression is high in earlier B cell ontogeny, but is down-regulated in post-germinal center plasma cells. Therefore amplification of the *DEK* gene may result in different expressional outcomes in different types of B cells and their progeny, depending on the cell-specific regulation of *DEK* expression that might be mediated via transcriptional, post transcriptional or translational mechanisms. Supporting our hypothesis, expression analysis of amplified genes in gliomas showed that not all amplified genes in these tumors are overexpressed and the repressed expression patterns of the genes in original (normal) tissue are maintained in the tumor tissue despite the amplification of the genes [26]. Similarly, we showed that different B-cell malignancies including BL, MZL and DLBCL show high level of DEK expression in malignant B cells (Fig 4), which was similar to their normal counterpart (Fig 3B).

Reduced DEK expression has distinct effects in different cell types. In epithelial cells, decreased DEK expression induces senescence and reduces tumor formation [7, 14], whereas it increases the number of myeloid progenitor cells in mice and stimulates myeloid colony formation *in vitro* [11]. In our study, stable knockdown or the overexpression of DEK in MM cell lines did not affect the proliferation or viability of the cells (Fig 6A and 6B). Similar to the literature [27], melphalan treatment of the MM cell lines induced cell death and cell cycle arrest, a process which was not affected by knockdown (Fig 6C and 6D) or overexpression of DEK (data not shown). Interestingly, knockdown of DEK in RPMI-8226 cells, which are already 98% positive for CD138 [28], resulted in a mild but significant increase in the expression level of this gene (Fig 7). All together, our results suggest a potential association between reduced DEK expression and level of CD138 expression on the plasma cells, which we aim in the future to further investigate using the primary plasma cell progenitors.

In conclusion, we have found a surprising down-regulation of DEK expression specifically in CD138^positive^ plasma cells, even in the setting of copy number gains of the *DEK* gene associated with neoplasia. Our findings suggest that high levels of DEK expression might be required for the proper proliferation of primary B cells whereas its downregulation might contribute to the development of terminally differentiated plasma cells. This hypothesis will be the subject of future research aiming to understand the role of *DEK* in normal and malignant plasma cell development.
